# The expression of EMX2 lead to cell cycle arrest in glioblastoma cell line

**DOI:** 10.1186/s12885-018-5094-y

**Published:** 2018-12-04

**Authors:** Annabelle Monnier, Rachel Boniface, Régis Bouvet, Amandine Etcheverry, Marc Aubry, Tony Avril, Véronique Quillien, Eric Chevet, Jean Mosser

**Affiliations:** 1 Univ Rennes, CNRS, IGDR [(Institut de génétique et développement de Rennes)]-UMR 6290, F-35000 Rennes, France; 2 CHU Rennes, Service de Génétique Moléculaire et Génomique Médicale, Rennes, France; 3 Univ Rennes, Plateforme GEH, CNRS, Inserm, BIOSIT - UMS 3480, US_S 018, F-35000 Rennes, France; 4 Univ Rennes, Inserm, CLCC Eugène Marquis, COSS [(Chemistry Oncogenesis Stress Signaling)]–UMR_S 1242, F-35000 Rennes, France

**Keywords:** Glioblastoma - reversible phenotype, Cell proliferation, Cell cycle arrest, Transcriptome analysis

## Abstract

**Background:**

Glioblastoma (GB) is a highly invasive primary brain tumor that nearly always systematically recurs at the site of resection despite aggressive radio-chemotherapy. Previously, we reported a gene expression signature related to tumor infiltration. Within this signature, the *EMX2* gene encodes a homeodomain transcription factor that we found was down regulated in glioblastoma. As *EMX2* is reported to play a role in carcinogenesis, we investigated the impact of *EMX2* overexpression in glioma-related cell lines.

**Methods:**

For that purpose, we constructed tetracycline-inducible *EMX2* expression lines. Transfected cell phenotypes (proliferation, cell death and cell cycle) were assessed in time-course experiments.

**Results:**

Restoration of EMX2 expression in U87 glioblastoma cells significantly inhibited cell proliferation. This inhibition was reversible after EMX2 removal from cells. EMX2-induced proliferative inhibition was very likely due to cell cycle arrest in G1/S transition and was not accompanied by signs of cell death.

**Conclusion:**

Our results suggest that EMX2 may constitute a putative therapeutic target for GB treatment.

Further studies are required to decipher the gene networks and transduction signals involved in EMX2’s effect on cell proliferation.

**Electronic supplementary material:**

The online version of this article (10.1186/s12885-018-5094-y) contains supplementary material, which is available to authorized users.

## Background

Glioblastoma (GB), a WHO grade IV glioma, is the most frequent and aggressive malignant primary brain tumor in adults [1, 2]. Its prognosis remains extremely dismal, despite multimodal standard treatment with surgery, radiotherapy, and temozolomide-based chemotherapy [3]. GB has been extensively characterized at the genomic, transcriptomic and epigenomic levels and is described as a molecularly heterogeneous disease at both inter- and intratumor levels [4–11]. Despite this heterogeneity, few prognostic and predictive biomarkers are clinically relevant [12, 13], and potentially in conjunction with this heterogeneity, new alternative therapeutic approaches have yet to prove their clinical utility.

*EMX2* encodes a homeodomain transcription factor, homologous to the *Drosophila melanogaster* empty spiracles (*ems*) [14]. It is primarily expressed in the urogenital tract, kidney [15] and brain [16]. EMX2 is essential for many aspects of cell growth and differentiation [17] and plays a key role during brain development by controlling several neurodevelopmental processes [16, 18]. EMX2 is expressed in the neural precursors of the subventricular brain zone [19]. It is involved in the regulation of differentiation and migration properties of neural precursor cells and acts in the transition between resident early progenitor and more mature precursors capable of migrating out of the subventricular zone [18]. EMX2 expression is reported to alter the proliferation of neural stem cells, thereby reducing their astrocytic outputs [20–22]. Thus, EMX2 may contribute to modulating the balance between neurogenesis and astrogenesis during brain development [23].

Several studies emphasize a tumor suppressor gene function for *EMX2*, initially suggested by its location within a genomic region of allelic loss in uterine endometrial adenocarcinoma [24, 25]. Further studies show that EMX2 suppresses cell proliferation and is down regulated in both endometrial and lung cancers [26–28]. Furthermore, EMX2 expression has documented prognostic and/or predictive values for survival of malignant pleural mesothelioma [29] and lung adenocarcinoma [30]. Recently, Falcone et al. reported that EMX2 overexpression suppresses GB growth both in vitro and in vivo, and induces apoptosis in GB cells [31].

Here, we confirm the anti-proliferative effect of EMX2 overexpression in GB cells. We show that this effect is reversible after EMX2 removal, does not lead to apoptosis in our experiments, and is very likely due to a G1 to S arrest in the cell cycle.

## Methods

### Cell culture

The experiments conformed to the principles set out in the WMA Declaration of Helsinki and the Department of Health and Human Services Belmont Report. GB samples were obtained after informed written consent from patients admitted to the neurosurgery department at Rennes University Hospital for surgical resection in accordance with the local ethic committee and with the French legislation or was obtained from the processing of biological samples through the Centre de Ressources Biologiques (CRB) Santé of Rennes BB-0033-00056. The research protocol was conducted under French legal guidelines and fulfilled the requirements of the local institutional ethics committee. Tumors used in this study were histologically diagnosed as grade IV astrocytoma according to WHO criteria. Adherent (RADH) and neurospheres (RNS) (enriched in stem cells) GB primary cell lines were obtained from GB samples as described in [32, 33]. Briefly, fresh tumor tissue was mechanically dissociated using gentleMACS dissociator following manufacturer’s instructions (Miltenyi Biotec). RADH cells were grown in Dulbecco’s Modification of Eagle’s Medium (DMEM, Lonza, Verviers, Belgium) supplemented with 10% fetal bovine serum (FBS) (Lonza). RNS cells were grown in DMEM/Ham’s: F12 (Lonza) supplemented with B27 and N2 additives (Invitrogen, Cergy Pontoise, France), EGF (20 ng/ml) and basic FGF (20 ng/ml) (Peprotech, Tebu-Bio). All GB RNS and RADH cells were used between the 5th and 15th passages for experiments. Human immortalized U251 MG (Sigma, St Louis, MO, USA) and U87 MG (ATCC) GB cell lines were cultured in DMEM 10% FBS.

### Construction of *EMX2* expression systems

pcDNA3.1/*EMX2* (NM_004098) mammalian expression-vector was sub cloned from pCMV6-XL5/*EMX2* vector (Origene). pcDNA3.1/*EMX2* and empty vector transfections were performed using Lipofectamine 2000 (cat. no. 11668072; Invitrogen; Thermo Fisher Scientific, Inc.) according to the manufacturer’s protocol. Transfected U87 GB cells were then transferred to T75-flasks for selection with G418 (2.5 mg/ml; Invitrogen). Stable transfectants were maintained in regular medium with G418 at 1 mg/ml concentration for further experiments.

**Fig. 1:**
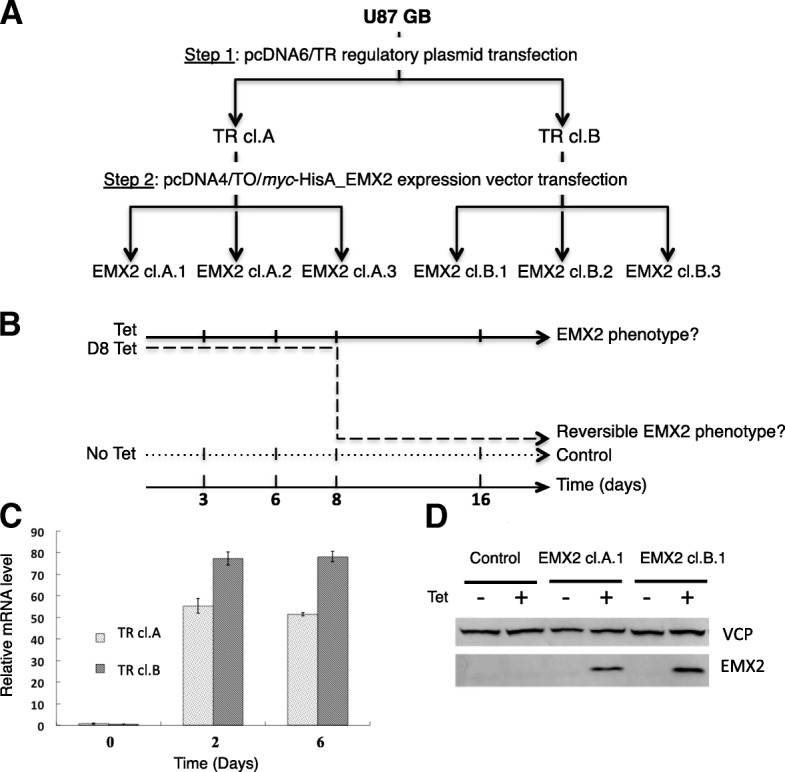
EMX2 expression in U87 transfected cells. **a**- Production of a tetracycline-regulated *EMX2* expression system in U87 cells. Experimental design. Six distinct, stable, double transfected clones were constructed. First, U87 cells were transfected using the regulatory vector pcDNA6/TR. The two resulting clones (TR cl. A and TR cl. B) were further transfected by means of the expression vector pcDNA4/TO/*myc-*HisA_*EMX2*. We selected three stable clones derived from each regulator clone, TR cl.A (EMX2 cl.A.1, EMX2 cl.A.2 and EMX2 cl.A.3) and TR cl.B (EMX2 cl.B.1, EMX2 cl.B.2 and EMX2 cl.B.3). **b**- Phenotype induced by EMX2 restoration. 16 days cell culture with or without EMX2 induction were carried out for the 6 clones described in A. This design allowed testing for (1) the *EMX2* overexpression phenotype (Tet); (2) the reversibility of this phenotype by Tet-induction arrest at day 8 (D8 Tet). Control conditions correspond to culture without tetracycline (No Tet). The same experiments were also performed using empty plasmid clones as described in Material and Methods. Complete sets of clones and associated conditions are depicted in Table A (Supplementary data) **c**-**d**- *EMX2* expression in the tetracycline-inducible system. *EMX2* mRNA levels in distinct clones: six independent clones were used (the three clones derived from the regulator clone TR cl.A and the three clones derived from the TR cl.B). *EMX2* mRNA level was measured at day 0 (no induction), day 2 and day 6 after tetracycline-induction (**c**). Welch Two Sample t-test on EMX2 cl.A. (J2 versus no Tet *p* = 0,00375; J6 versus no Tet p = 0,00008) and on EMX2 cl.B. (J2 versus no Tet p = 0,00161; J6 versus no Tet p = 0,00091). EMX2 protein levels after six days of culture with (+) or without (−) tetracycline-induction in empty clones (Control) and two independant EMX2 clones (**d**).

T-Rex Tet-On System (Invitrogen) was used to produce a tetracycline-regulated *EMX2* expression system. U87 cells were transfected with a regulatory plasmid (pcDNA6/TR), which encodes the tetracycline (Tet) repressor. Individual clones were expanded using blasticidin selection (5 μg/ml; Invitrogen) and tested for Tet induction (1 μg/ml, Sigma-Aldrich) by transient transfection with a *lacZ* gene in a control inducible plasmid. Two clones, TR cl.A and TR cl.B, were selected as they displayed very low background *lacZ* expression and strong *lacZ* induction by Tet. pcDNA4/TO/*myc*-HisA_*EMX2* mammalian expression vector was sub cloned from the pCMV6-XL5/*EMX2* vector. Next, TR cl.A and TR cl.B stable clones were transfected with pcDNA4/TO/*myc-*HisA_*EMX2*. Individual resulting double transfected clones were expanded in presence of blasticidin (3 μg/ml; Invitrogen) and zeocin (20 μl/ml; Invitrogen), and further tested for *EMX2* expression in response to Tet. Three individual clones derived from TR cl.A (EMX2 cl.A.1, EMX2 cl.A.2, EMX2 cl.A.3) and three individual clones obtained from TR cl.B (EMX2 cl.B.1, EMX2 cl.B.2, EMX2 cl.B.3) were selected as they displayed high *EMX2* expression in response to Tet and very low background *EMX2* expression level in absence of Tet. Two stable lines expressing an empty vector were used as controls (empty cl.A and empty cl.B) (See Fig. 1 for experimental design).

The same experimental design was performed on U251 GB cells. Briefly, six distinct stable double transfected clones were constructed. First, U251 cells were transfected using the regulatory vector pcDNA6/TR. The two resulting clones (TR cl. C and TR cl. D) were further transfected with the expression vector pcDNA4/TO/*myc-*HisA_*EMX2*. Individual resulting double transfected clones were expanded in presence of blasticidin (5 μg/ml) and zeocin (50 μl/ml). We selected three stable clones derived from each regulator clone, TR cl.C (EMX2 cl.C.1, EMX2 cl.C.2 and EMX2 cl.C.3) and TR cl.D (EMX2 cl.D.1, EMX2 cl.D.2 and EMX2 cl.D.3) (Additional file 1: Figure S1a).

### Cell proliferation assay

Cell proliferation was measured using a WST-1 cell proliferation reagent (Roche Applied Science). Cells were plated in 96 well plates (1.10^3^ cells/well) and cultured for one to 28 (U87) or 31 (U251) days in the presence or absence of Tet (1 μg/ml). Proliferation measures were performed at the following days: 0, 1, 2, 3, 6, 9, 13, 16, 20, 23, 28 and 31. At each time point, 20 μl/well WST-1 (1:10 final dilution) reagent was added, and cells were incubated at 37 °C for four hours following optical density measurement at 450 nm. All experiments were performed in triplicate.

### Apoptosis assays

#### Flow cytometry analysis

Cells were seeded in T75-flasks (4.10^5^ cells/flask) and cultured for three and seven days in the presence or absence of Tet (1 μg/ml). At each time point, cells were harvested and stained with Annexin V-PE and 7-AAD (BD Pharmingen) according to the manufacturer’s instruction. The fraction of apoptotic cells was quantified using a BD FACSCanto II system (BD Biosciences).

#### Caspase-3 catalytic activity assay

Cells were seeded in T25-flasks (2.10^5^ cells/flask) and cultured for one, three and seven days in the presence or absence of Tet (1 μg/ml). At each time point, cells were harvested and Caspase-3 activity was determined using the Caspase 3/CPP32 Colorimetric Assay kit, according to the manufacturer’s instructions (Biovision, Inc., Milpitas, CA, USA). Briefly, the cells were lysed in caspase 3 sample lysis buffer (Biovision, Inc.). The homogenates were then centrifuged at 10,000×g and 4 °C for 1 min and the supernatant was collected for protein estimation using Bradford assay. 200 μg of total protein were then exposed to the DEVD substrate conjugate provided in the kit for 4 h at 37 °C. The sample was measured in an automatic microplate reader at 405 nm. At each time point, a positive control is achieved by addition of staurosporine (10 μM – Sigma) 24 h prior to measurement of caspase activity.

### Cell cycle analysis

Cells were seeded in T75-flasks (0.25 X 10^6^ cells/flask) and cultured for three and seven days in presence or absence of Tet (1 μg/ml). At each time point, cells were harvested and analyzed by flow cytometry with propidium iodide staining using BD Cycletest Plus DNA kit (BD Biosciences) according to the manufacturer’s instructions. The percentage of cells in each cell cycle phase was measured by flow cytometry on BD FACSCanto II (BD Biosciences) and analyzed using Modfit LT v4.1.7 software (Verity Software House).

### Real-time quantitative RT-PCR (RT-qPCR) analysis

Total RNA was extracted from cells using NucleoSpin RNAII kit (Macherey-Nagel) according to the manufacturer’s instructions. cDNA was synthesized using the Primescript RT reagent Kit (Takara). Quantitative real-time PCR was performed using Power SYBR® Green PCR Master Mix (Applied Biosystems). All primer sequences used are shown in supplementary Table B. Relative expression levels of the target mRNA were calculated using 2 ^-ΔΔCT^ method [34] and were normalized to *GAPDH* and *TBP* reference genes.

### Western blot analysis

Total protein was extracted from cells using extraction solution (50 mM Tris pH 7.4, 250 mM NaCl, 1 mM EDTA, 1% NP40 and 1 mM DTT) supplemented with protease inhibitors (Thermo Fisher Scientific, Inc.). Samples were incubated on ice for five minutes followed by centrifugation (1700 rpm, 4 °C, 5 min). Protein concentrations were determined using the Bradford method (Pierce Coomassie Protein Assay Kit, Life Technologies). Samples (20 μg protein/lane) were separated on 10–12% SDS-PAGE gels and transferred onto nitrocellulose membranes (Amersham). After blocking in PBS-Tween 0.1% buffer containing 5% non-fat milk, membranes were first incubated with mouse monoclonal anti-MYC antibody (diluted 1:5000, Invitrogen), Cyclin B1 (diluted 1:10000, Abcam) or mouse monoclonal anti-VCP antibody (diluted 1:7000, BD Biosciences) for 2 h following incubation with goat polyclonal anti-mouse Immunoglobulins/HRP secondary antibodies (diluted 1:7000, Dako) for one hour. Subsequently, blots were imaged using an enhanced chemiluminescence kit (Amersham).

### Transcriptome analysis

Transcriptome profiling was performed on the three EMX2 cl.A clones (EMX2 cl.A.1, EMX2 cl.A.2 and EMX2 cl.A.3) at day 2 (D2 Tet), day 6 (D6 Tet) and day 16 (D16 Tet) after Tet induction. The corresponding non-induced conditions were used as controls (No Tet). To test reversibility of the Tet-induced phenotype, we also included a day 16 condition with or without an arrest of Tet induction at day 8 (D8 Tet and D16 test, respectively). Each condition was tested in triplicate (biological replicates). For test point of this time-course experiment, we also tested the transcriptome of the empty expression vector cells (Additional file 1: Table S1).

Total RNA was isolated using the NucleoSpin RNAII Kit (Macherey-Nagel). RNA integrity (RNA Integrity Number ≥ 8) was confirmed with an Agilent 2100 bioanalyzer (Agilent Technologies). Gene expression profiling was carried out with the Agilent whole human genome 8x60K microarray kit (Agilent Technologies). Total RNA was extracted, labeled and hybridized according to kit manufacturer’s recommendations. Raw intensity data were log2-transformed and normalized (intra-array and inter-array scaling) using GeneSpring GX software (Agilent Technologies). Gene expression data are shown in Additional file 1: Appendix A.

Probes were pre-selected according to their level of expression (above background) and to their level of variation between samples (sd ≥ 50%). Two-way analysis of variance (ANOVA) using ‘day’ and ‘Tet-induction’ as factors was performed to identify Tet-induced probes. Adjusted *p* values were calculated by controlling for the false discovery rate using the Benjamini & Hochberg procedure. Probes were considered significantly differentially expressed (DE) if the adjusted p value was below 1.10^− 3^.

We used Gene Set Enrichment Analysis (GSEA) to identify enriched molecular signatures among DE genes [35]. A gene set was considered significantly enriched if the Fisher Exact test q value was below 1.10^− 3^.

### Statistical modeling of cell proliferation assay

Statistical analysis consisted of testing the optical density (OD, a measure of proliferation) against time (a covariate), TET (a fixed factor with three levels: No Tet, Tet and D8 Tet) and the clone (a fixed factor with two levels: empty vector or clone with EMX2). Interactions between the three explanatory variables were considered in the models to test whether temporal dynamics of the optical density (OD) varied between TET-levels and/or clone-levels. In our experimental set-up, a given subject was measured repeatedly over time. To recognize a correlation between serial observations on the same subject, the subject ID was implemented in the statistical model as a random effect. We considered a first-order autoregressive (AR1) correlation structure for the model residuals (i.e., observations close in time are more correlated than observations further apart). From plotting optical density over time, a quadratic relationship between both variables was suggested. Then, we fitted polynomial (i.e., quadratic model: *y* = *β*_*0*_ + *β*_*1*_.*x* + *β*_*2*_.*x*^2^) models to the set of points and retained the polynomial shape of the relationship if the quadratic term of the model that represents the amount of curvature in the data (i.e., *β*_*2*_) was significantly different from zero. For statistical modeling, the R package ‘*nlme*’ [36] was used. Estimation of model parameters was based on restricted maximum likelihood (REML). Residuals of fitted models were visually inspected (quantile-quantile plot and residuals vs. predicted values plot), and all were approximately normal, i.e., unimodal and symmetric.

## Results

### *EMX2* expression is low in GB and GB cell lines

**Fig. 2:**
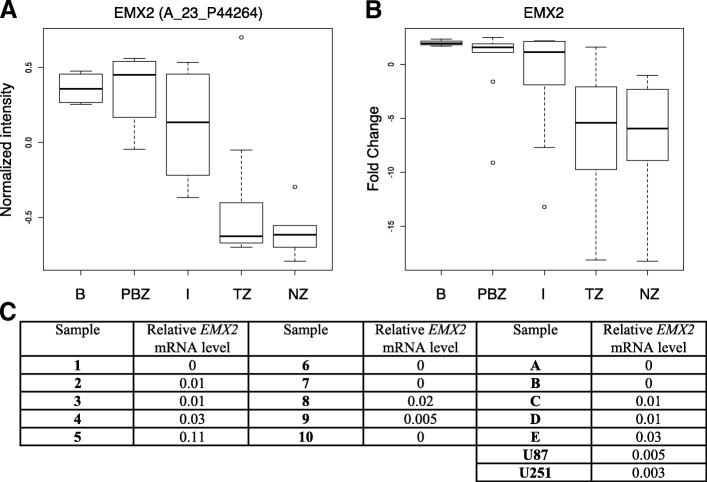
*EMX2* expression in GB patients and cell lines. **a**-**b**- Intratumor *EMX2* expression in patients. *EMX2* mRNA level was measured at four different GB zones from 10 patients (PBZ: peripheral brain zone, I: interface zone, TZ: tumor zone, and NZ: necrotic zone; [10]) and on two normal brain used as control (**b**: normal brain). Box plots representing mRNA expression levels obtained from microarray experiments for the *EMX2* A_23_P44246 Agilent probe (**a**). *p* = 1.573e-06. *EMX2* mRNA level was measured by RT-qPCR. Box plots representing fold changes calculated using RNA brain as sample reference and *GAPDH* and *TBP* as gene reference (**b**). *p* = 1.38e-02. **c**- *EMX2* expression level in GB cell lines. RT-qPCR was used to measure EMX2 mRNA level in ten primary adherent cell cultures (1–10), five neurosphere cultures (**a**-**d**) and two immortalized GB cell lines U87 and U251. Relative *EMX2* expression level was normalized to *GAPDH* and *TBP* reference genes and to a RNA brain reference sample. Experiments were performed in triplicate. Since relative *EMX2* expression level was extremely low or null, the standard deviations could not be indicated in this table

To assess *EMX2* mRNA expression level, we focused on a previously reported transcriptome gene signature associated with GB intratumor heterogeneity (published by Aubry et al. [10] We observed a significant decrease of *EMX2* expression in GB tumor and necrotic zones compared to paired interface and peripheral brain zones derived from the same patients (*n* = 10) (Fig. 2a). We confirmed *EMX2* expression profile by performing RT-qPCR on RNA samples from the same-paired GB zones (Fig. 2b). We further measured *EMX2* expression by RT-qPCR in different GB cell lines: ten primary adherent cell lines, five neurosphere cell lines, and two immortalized cell lines. As expected, *EMX2* expression was low or null in each case (Fig. 2c).

### Tetracycline-induced *EMX2* expression in U87 GB cell line

As we never obtained stable EMX2 expression clones in U87 cells, we generated a Tet-inducible system and selected six distinct tetracycline-inducible EMX2 clones: (1) three double transfected clones derived from one unique Tet repressor transfected clone (EMX2 cl.A.1, EMX2 cl.A.2, EMX2 cl.A.3) and (2) three double transfected clones derived from a second independent Tet repressor clone (EMX2 cl.B.1, EMX2 cl.B.2, EMX2 cl.B.3) (Fig. 1a-b). We validated the efficiency of Tet induction by measuring EMX2 mRNA and protein levels in the presence or in absence of Tet (Fig. 1c-d).

### *EMX2* expression blocks GB cell proliferation in vitro

**Fig. 3:**
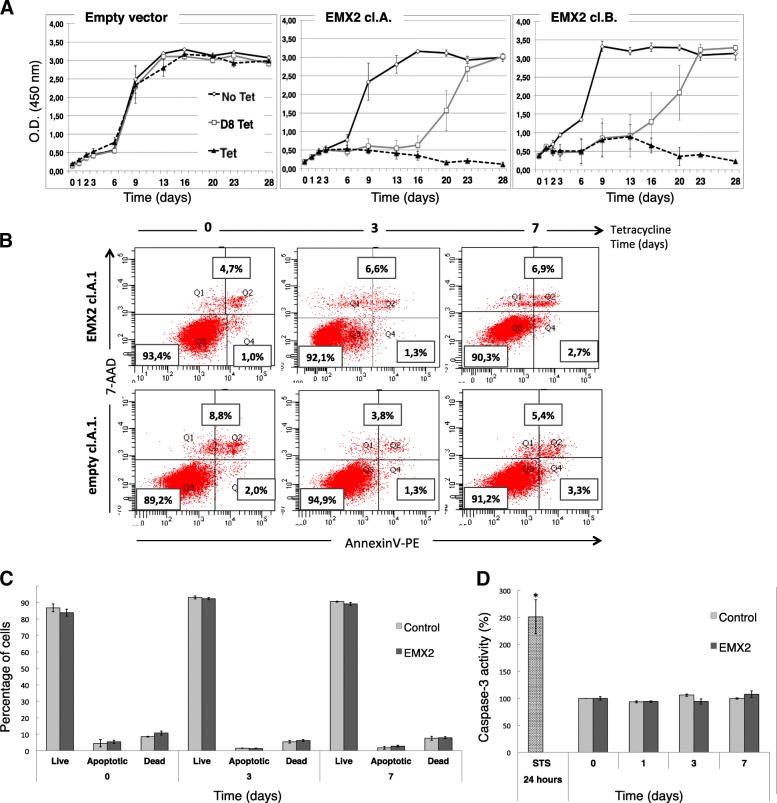
EMX2 induces a reversible proliferation block in U87 GB transfected cells. **a**- Cell proliferation was measured in three different culture conditions over 28 days without tetracycline (No Tet-black line), with tetracycline (Tet-dotted line) and with tetracycline for the first 8 days only (D8 Tet-gray line). Distinct triplicate experimental cultures were performed for empty vector and for all EMX2 cl.A or EMX2 cl.B (see Fig. 2). Proliferation curves were statistically validated using a linear mix model (see Materials and Methods) **b**-**c**- Cell death was assessed before (time 0), 3 and 7 days after addition of tetracycline for empty clones (Control) and EMX2 cl.A clones (EMX2). After treatment, cells were stained with AnnexinV-PE and 7-AAD then analyzed by a FACscan flow cytometer. The labeling patterns identify different cell populations: vital cells (7AAD-negative/ annexin V-negative) in region Q3, apoptotic cells (7AAD-negative/annexinV-positive) in region Q4, dead cells (7AAD-positive/annexin V-positive) in region Q2 and damaged cells (7AAD-positive/annexin V-negative) in region Q1. Cell percentages are shown for Q3, Q4 and Q1 + Q2 regions (**b**). A representative histogram for viability, apoptosis and necrosis is shown in (**c**). Data are expressed as mean ± SE. Experiment was carried out in triplicates using three distinct clones. *p* value is not significant compared to control group. Effect of EMX2 overexpression on apoptosis in U87 GB cells (**d**). Caspase-3 activity was measured in cell lysates after 1, 3 and 7 days after Tet induction. Each column represents the mean value (^+^ SD) of three independent clones (*n* = 3, Student’s test) normalized to non-treated cells (taken as 100%). Positive controls were performed at each time point (0, 1, 3 and 7 days) and consisted of adding staurosporine (10 μM) 24 h before Caspase-3 activity dosage normalized to non-treated cells (taken as 100%) (*n* = 12, Student’s test, * *p* < 0.001).

Tet-induced *EMX2* expression in U87 GB cells led to a significant decrease in cell proliferation, whereas cell proliferation was unaffected in the absence of Tet. Interestingly, when Tet induction was stopped at day 8, proliferation level was restored, suggesting a reversible phenotype (Fig. 3a). To confirm this phenotype induced by EMX2 we performed the same experiment using another glioblastoma cell line: U251. Indeed, we observed that overexpression of EMX2 in three different double transfected clones induced a reversible cell cycle arrest (Additional file 1: Figure S1d).

### *EMX2* expression does not induce apoptosis in vitro

We next tested for apoptosis and necrosis phenotypes during a period of 7 days after Tet induction. No evidence of apoptosis and/or necrosis was observed (Fig. 3b-d). This is consistent with the reversibility of proliferation inhibition induced by EMX2 (Fig. 3a).

### Transcriptome of U87 GB cells overexpressing *EMX2*

**Fig. 4:**
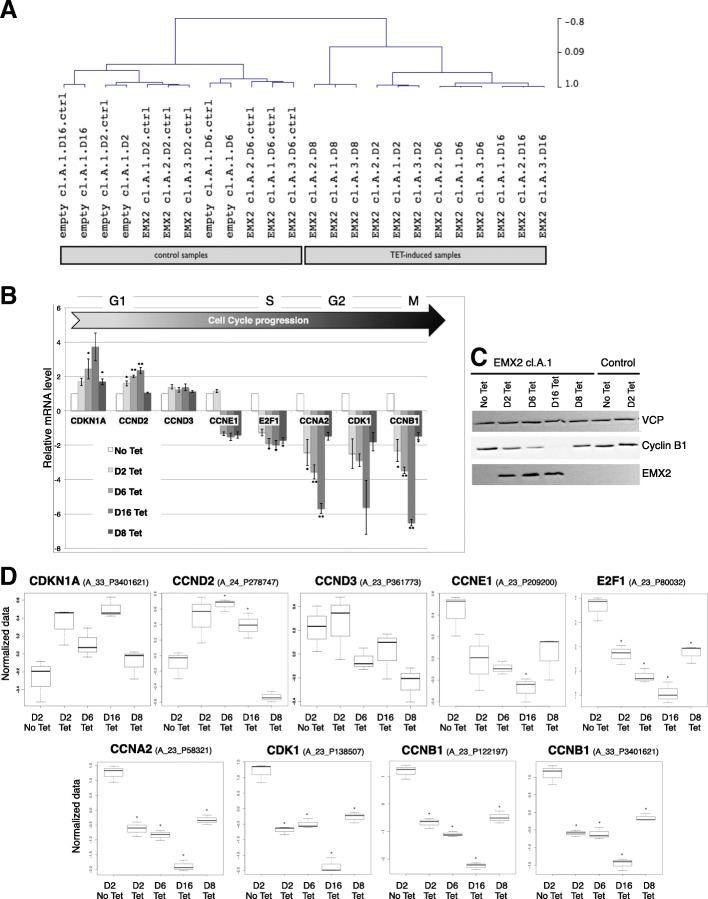
EMX2 restoration in U87 GB cells induces G1/S transition arrest. **a**-Sample clustering based on differentially expressed genes in Tet-induction (3916 DE probes). Pearson correlation distance is indicated on the right of the dendrogram. **b**-RT-qPCR was used to measure mRNA levels for genes associated with cell-cycle phases. Experiments were performed on the three EMX2 cl.B clones (EMX2 cl.B.1, EMX2 cl.B.2 and EMX2 cl.B.3). **c**-Western blotting experiment on EMX2 cl.A.1 and on control (empty vector) recombinant U87 cells using EMX2 and Cyclin B1 antibodies. VCP served as a loading control. Cells were grown in the absence (No Tet) or presence of tetracycline for 2 days (D2 Tet), 6 days (D6 Tet), 16 days (D16 Tet), and 8 days with following 8 days without tetracycline (D8 Tet) to test the induced phenotype and its reversibility. T-tests were performed using the No Tet condition as the reference and *p*-values were adjusted with BH correction. * *p* < 0.05, ** *p* < 0.01. **d**- Box plots representing mRNA expression normalized to levels obtained by microarray experiments for indicated probes of cell cycle-related genes. Transcriptome experiments were performed on the three EMX2 cl.A clones (EMX2 cl.A.1, EMX2 cl.A.2 and EMX2 cl.A.3). The differences between control conditions compared to No Tet were evaluated by t-test and corrected with BH. * *p* < 0.05, ** *p* < 0.01

Of the 11,253 pre-selected probes, 3916 were differentially expressed (DE) between control and Tet-induced conditions (Additional file 1: Appendix A). Hierarchical clustering of the DE probes (Pearson correlation, average distance) discriminated samples within two groups: (1) a group of control samples including empty vector conditions and non-induced control conditions, and (2) a group of Tet-induced conditions (Fig. 4a). Interestingly, in Tet-induced conditions the DE genes discriminated between day 8 and day 16 conditions (Fig. 4a). Nevertheless, the day 8 condition did not segregate with the group of control samples, suggesting that proliferation reversibility is initiated but not completely established at day 16 (according to proliferation curves displayed on Fig. 3a).

Functional annotation of the 2519 corresponding DE genes identified as significantly enriched Gene Ontology (GO) biological processes, among them: *cell cycle* (*n* = 345 genes), *chromosome organization* (*n* = 271), *cellular response to stress* (*n* = 315) and *RNA processing* (*n* = 217) (Additional file 1: Appendix A). When restricting GSEA to the 345 GO *cell cycle* associated genes, we found significantly enriched Reactome canonical pathways, among them: DNA replication (n-83 genes), *mitotic_M_M_G1_phases* (*n* = 78 genes), and *mitotic_G1_G1_S_phases* (*n* = 46 genes) (Additional file 1: Appendix A). In our transcriptome data, we observed that genes involved in M/G1 phase transition and in G1/S phase transition were downregulated in Tet-induced cells. To further query these results, we performed cell cycle analysis.

### EMX2 may reversely block the cell cycle at G1/S transition

Transcriptome data strongly suggested that cell proliferation blockage might involve genes associated with M/G1 and/or G1/S phases. Therefore, we measured mRNA levels of selected genes involved in different cell cycle phases or checkpoints in U87 cells transfected with the EMX2 cl.B clones (Fig. 4b). Consistent with our transcriptome data (obtained for both clones cl.A and cl.B), *CDKN1A* and *CCND2* (involved in early G1 phase) were upregulated when *EMX2* was expressed. CDKN1A (cyclin-dependent kinase inhibitor 1A; also known as p21, Cip1, Waf1) is a tumor suppressor that regulates cell proliferation [37] and binds to and inhibits kinase activity of Cdk2 and Cdk1, leading to cell cycle arrest. CCND2 has a critical role in cell cycle progression, especially in glioblastoma stem cells, and causes G1 arrest in vitro in GSC cells [38].

In contrast, *E2F1* and *CCNA2* were downregulated when *EMX2* was expressed. E2F1 is considered one of the major proteins responsible for entry into S phase and cell cycle progression [39]. Cyclin A2 expression is synchronized during the cell cycle and regulated by E2F in G1 phase. Cyclin A2-CDK complexes are likely to be important for continued DNA synthesis and progression into G2 phase in somatic dividing cells [40, 41]. The strong down regulation of cyclin A2 observed in our experiments may lead to inhibition of initiation and progression of DNA synthesis and S phase.

Moreover, *CDK1* and *CCNB1* were also downregulated when *EMX2* was expressed. The cyclin-dependent kinase CDK1 is known to control the G2/M transition though binding with cyclin B1. CDK1 is now recognized as the only essential cell cycle CDK. CDK1 is equally capable of promoting G1/S transition by binding to Cyclins E and A. Even in the presence of CDK2, CDK1 may still be the prominent kinase at G1/S [42–44]. Therefore, the strong repression of CDK1 expression may also contribute to the observed cell cycle arrest at the G1/S transition.

All these results taken together confirm that *EMX2* expression leads to a late G1 phase or G1/S transition arrest.

Again, the reversibility of the proliferation inhibition was observed at the mRNA expression level for all selected genes (Fig. 4b) and at the protein level for Cyclin B1 (CCNB1) (Fig. 4c). Indeed Tet-induction arrest led to inversion of their expression levels.

### EMX2 induces reversible S phase cell cycle arrest

**Fig. 5:**
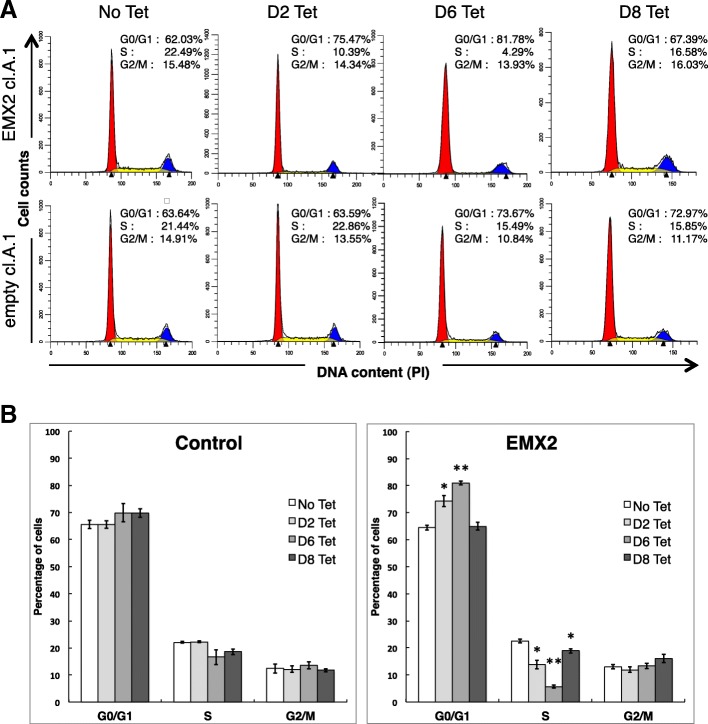
EMX2 overexpression strongly diminished S phase of the cell cycle in U87 cells. Cell number distribution among cell cycles phases was measured for three empty clones (Control) and six distinct EMX2 clones (EMX2 cl.A.1, EMX2 cl.A.2 and EMX2 cl.A.3, EMX2 cl.B.1, EMX2 cl.B.2 and EMX2 cl.B.3). Cells were cultured without (No Tet) or with tetracycline for 2 days (D2 Tet), 6 days (D6 Tet), or 8 days followed by 8 days of culture without tetracycline (D8 Tet). After treatment, cells were stained with propidium iodide then analyzed by a FACscan flow cytometer. The peaks in the illustration correspond to the G0/G1, S and G2/M phases (**a**). Percentages in each phase of the cell cycle were automatically measured by Modfit LT. A representative histogram of cell cycle distribution is shown in **b**. Data are expressed as mean ± SE. The No Tet condition was used as reference in the t-tests and p-values were adjusted with BH correction * *p* < 0.05, ** *p* < 0,01

To differentiate between G2/M and G1/S cell cycle phase arrests, we performed flow cytometry analysis (Fig. 5). The number of cells in the S phase was significantly reduced by Tet-induced EMX2 expression. When EMX2 induction was stopped at day 8, cell number at S phase was restored to initial levels. This observation agrees with a reversible G1/S phase arrest by EMX2 restoration in U87 GB cells.

## Discussion

In the present study, we report that *EMX2* expression is highly decreased in GB tumor zones relative to matched adjacent normal brain zones from the same patients (Fig. 2a). We also observed that *EMX2* is barely expressed in immortalized GB or primary cell lines (Fig. 2c). Our results suggest that *EMX2* expression in the U87 GB cell line induces a G1/S cell cycle arrest resulting in decreased cell proliferation. We further show that this inhibition is reversible after stopping EMX2 expression. These findings are supported by an original and robust experimental design (two glioblastoma cell lines, six distinct inducible EMX2 clones originating from two independent regulator clones for each cell line) and a multimolecular level analysis (RNA, protein and cells).

Our results are in agreement with reported descriptions of EMX2 as a protein implicated in the progression of some cancers. Indeed, EMX2 has a documented anti-proliferative effect in vitro and anti-tumorigenesis effect in vivo in some solid cancers [28, 45–47]. In GB cell culture, Falcone et al. reported that proliferation inhibition induced by EMX2 was associated with cell collapse and apoptosis activity within 7–8 days [31]. We did not observe enhanced cell death until 7 days of culture, and we observed no sign of apoptosis over 16 days of culture. Furthermore, transcriptome analysis showed that EMX2 overexpression did not modify the expression of cell death annotated genes. These phenotypic differences may result from experimental procedures that differ in transfection and induction processes.

A major and original finding from this study is that EMX2’s anti-proliferative effect is significantly reversible after 8 days of induction in U87 and U251 glioblastoma cell lines. This phenotype reversibility was indeed observed or strongly suggested in all our experiments: (1) cell culture experiments clearly demonstrated a cell proliferation blockage induced by *EMX2* overexpression and a proliferation restart after *EMX2* expression arrest (Fig. 3a, Additional file 1: Figure S1d), (2) transcriptome analysis suggested that proliferation reversibility is initiated but not completely established at day 16 (Fig. 4a), (3) cell cycle cytometry analysis showed a significant reduction of the number of cells in S phase following *EMX2* overexpression and a return to initial level when *EMX2* expression is blocked (Fig. 5), and (4) expression profiles at both the RNA and protein levels of selected cell cycle genes also suggested a reversible G1/S cell cycle arrest (Fig. 4b-c). Reversibility is a key element when considering inhibition of EMX2 as a potential therapeutic strategy. The reversibility of cell cycle arrest by EMX2 seems to be important in the tissue development but although in tumorogenesis. Very recently Lee et al. reported that IDH wild-type glioblastoma originates from subventricular zone cells that present low level driver mutations [48]. Interestingly EMX2 is expressed in neural progenitor cells located in the subventricular zone. We can hypothesize that EMX2 may control the transition (plasticity) between cancer glioblastoma stem cells and tumor more differentiated cells and, by this way, influence the GBM recurrence after surgery.

We clearly show that EMX2 induction leads to variation of cell cycles genes at the mRNA and/or protein levels (Fig. 4-B-C). However, EMX2’s mechanism of action in glioblastoma remains unclear. Inhibition of Wnt signaling by EMX2 has been proposed to block the cell cycle in some cancers [30, 45, 46]. In our transcriptome experiment, beta-catenin was not down regulated by EMX2. Only one of the beta-catenin target genes, *MYC* was found to be down regulated by EMX2 overexpression.

## Conclusion

Further studies are required to explore the link between EMX2 expression and the Wnt signaling pathway in the regulation of cell cycle in GB. Besides, combined analysis of *EMX2* induced transcriptome and *EMX2* ChIP-Seq experiments may be greatly useful for deciphering gene networks and signaling pathways induced by the restoration of *EMX2* expression, a tumor suppressor gene in GB.

## Additional file

Additional file 1: (DOCX 318 kb)

## Abbreviations

CCN: Cyclin
CDK: Cyclin Dependant Kinase
ChIP-Seq: Chromatin immunoprecipitation (ChIP) sequencing
*E2F1*: *E2F Transcription Factor 1*
*EMX2*: *Empty Spiracles Homeobox 2*
*GAPDH*: *Glyceraldehyde-3- Phosphate Dehydrogenase*
GB: Glioblastoma
RT-qPCR: Real Time quantitative RT-PCR
TBP: TATA-Box Binding Protein
TET: Tetracycline
