# Supplementary material for: The expression of EMX2 lead to cell cycle arrest in glioblastoma cell line
Source: BMC Cancer. 2018 Dec 4;18:1213. doi: 10.1186/s12885-018-5094-y (PMC6280364; doi:10.1186/s12885-018-5094-y)
Supplement: Supplementary file 1 — (DOCX 318 kb) [file 12885_2018_5094_MOESM1_ESM.docx]

**Additional file**

| Condition name | Day 2 | | Day 6 | Day 8 | Day 16 | Condition name | Day 2 | Day 6 | Day 8 | Day 16 |
| --- | --- | --- | --- | --- | --- | --- | --- | --- | --- | --- |
| EMX2 conditions | | | | | | | | | | |
| EMX2 cl.A.1.D2 * | + | |  |  |  | EMX2 cl.B.1.D2 ❖ | + |  |  |  |
| EMX2 cl.A.1.D2.ctrl * | - | |  |  |  | EMX2 cl.B.1.D2.ctrl ❖ | - |  |  |  |
| EMX2 cl.A.2.D2 * | + | |  |  |  | EMX2 cl.B.2.D2 ❖ | + |  |  |  |
| EMX2 cl.A.2.D2.ctrl * | - | |  |  |  | EMX2 cl.B.2.D2.ctrl ❖ | - |  |  |  |
| EMX2 cl.A.3.D2 * | + | |  |  |  | EMX2 cl.B.3.D2 ❖ | + |  |  |  |
| EMX2 cl.A.3.D2.ctrl * | - | |  |  |  | EMX2 cl.B.3.D2.ctrl ❖ | - |  |  |  |
| EMX2 cl.A.1.D6 * | + | | + |  |  | EMX2 cl.B.1.D6 ❖ | + | + |  |  |
| EMX2 cl.A.1.D6.ctrl * | - | | - |  |  | EMX2 cl.B.1.D6.ctrl ❖ | - | - |  |  |
| EMX2 cl.A.2.D2 * | + | | + |  |  | EMX2 cl.B.2.D2 ❖ | + | + |  |  |
| EMX2 cl.A.2.D6.ctrl * | - | | - |  |  | EMX2 cl.B.2.D6.ctrl ❖ | - | - |  |  |
| EMX2 cl.A.3.D6 * | + | | + |  |  | EMX2 cl.B.3.D6 ❖ | + | + |  |  |
| EMX2 cl.A.3.D6.ctrl * | - | | - |  |  | EMX2 cl.B.3.D6.ctrl ❖ | - | - |  |  |
| EMX2 cl.A.1.D16 * | + | | + | + | + | EMX2 cl.B.1.D16 ❖ | + | + | + | + |
| EMX2 cl.A.1.D8 * | + | | + | + | - | EMX2 cl.B.1.D8 ❖ | + | + | + | - |
| EMX2 cl.A.1.D16.ctrl | - | | - | - | - | EMX2 cl.B.1.D16.ctrl | - | - | - | - |
| EMX2 cl.A.2. D16 * | + | | + | + | + | EMX2 cl.B.2. D16 ❖ | + | + | + | + |
| EMX2 cl.A.2.D8 * | + | | + | + | - | EMX2 cl.B.2.D8 ❖ | + | + | + | - |
| EMX2 cl.A.2.D16.ctrl | - | | - | - | - | EMX2 cl.B.2.D16.ctrl | - | - | - | - |
| EMX2 cl.A.3. D16 * | + | | + | + | + | EMX2 cl.B.3. D16 ❖ | + | + | + | + |
| EMX2 cl.A.3.D8 * | + | | + | + | - | EMX2 cl.B.3.D8 ❖ | + | + | + | - |
| EMX2 cl.A.3.D16.ctrl | - | | - | - | - | EMX2 cl.B.3.D16.ctrl | - | - | - | - |
| Empty clones conditions | | | | | | | | | | |
| empty cl.A.1.D2 * | + |  | |  |  | empty cl.B.1.D2 ❖ | + |  |  |  |
| empty cl.A.1D2.ctrl * | - |  | |  |  | empty cl.B.1.D2.ctrl ❖ | - |  |  |  |
| empty cl.A.2.D2 | + |  | |  |  | empty cl.B.2.D2 | + |  |  |  |
| empty cl.A.2.D2.ctrl | - |  | |  |  | empty cl.B.2.D2.ctrl | - |  |  |  |
| empty cl.A.3.D2 | + |  | |  |  | empty cl.B.3.D2 | + |  |  |  |
| empty cl.A.3.D2.ctrl | - |  | |  |  | empty cl.B.3.D2.ctrl | - |  |  |  |
| empty cl.A.1.D6 * | + | + | |  |  | empty cl.B.1.D6 | + | + |  |  |
| empty cl.A.1.D6.ctrl * | - | - | |  |  | empty cl.B.1.D6.ctrl | - | - |  |  |
| empty cl.A.2.D6 | + | + | |  |  | empty cl.B.2.D6 | + | + |  |  |
| empty cl.A.2.D6.ctrl | - | - | |  |  | empty cl.B.2.D6.ctrl | - | - |  |  |
| empty cl.A.3.D6 | + | + | |  |  | empty cl.B.3.D6 | + | + |  |  |
| empty cl.A.3.D6.ctrl | - | - | |  |  | empty cl.B.3.D6.ctrl | - | - |  |  |
| empty cl.A.1.D16 * | + | + | | + | + | empty cl.B.1.D16 | + | + | + | + |
| empty cl.A.1.D16.ctrl * | - | - | | - | - | empty cl.B.1.D16.ctrl | - | - | - | - |
| empty cl.A.2.D16 | + | + | | + | + | empty cl.B.2.D16 | + | + | + | + |
| empty cl.A.2.D16.ctrl | - | - | | - | - | empty cl.B.2.D16.ctrl | - | - | - | - |
| empty cl.A.3.D16 | + | + | | + | + | empty cl.B.3.D16 | + | + | + | + |
| empty cl.A.3.D16.ctrl | - | - | | - | - | empty cl.B.3.D16.ctrl | - | - | - | - |

Table S1: Summary of clone names and corresponding tetracycline induction times in culture conditions. (*): conditions used for transcriptome experiments. (❖): conditions used for RT-qPCR validation of transcriptome data.

| **Gene Symbol** | **Forward (5' 3') primer** | **Reverse (3' 5') primer** |
| --- | --- | --- |
| TBP | GAGCTGTGATGTGAAGTTTCC | TCTGGGTTTGATCATTCTGTAG |
| GAPDH | AGATCCCTCCAAAATCAAGTGG | GGCAGAGATGATGACCCTTTT |
| EMX2 | CACTAGCCCCGAGAGTTTCC | GGCGTGTTCCAGCCTTAGAA |
| CCNA2 | GGTACTGAAGTCCGGGAACC | GAAGATCCTTAAGGGGTGCAA |
| CCNB1 | CATGGTGCACTTTCCTCCTT | AGGTAATGTTGTAGAGTTGGTGTCC |
| CCND2 | CCGACAACTCCATCAAGCCT | ACTTAAAGTCGGTGGCACACA |
| CCND3 | GGGATCACTGGCACTGAAG | CCTGAGGCTCTCCCTGAGT |
| CCNE1 | GGCCAAAATCGACAGGAC | GGGTCTGCACAGACTGCAT |
| CDK1 | TGGATCTGAAGAAATACTTGGATTCTA | CAATCCCCTGTAGGATTTGG |
| CDKN1A | CCGAGGCACTCAGAGGAG | AGCTGCTCGCTGTCCACT |
| E2F1 | AAGTCCAAGAACCACATCCAGT | CTGGGTCAACCCCTCAAG |

Table S2: Sequence of primers used for gene amplification in RT-qPCR experiments

Appendix A: The 3,916 probes selected by two-way ANOVA (Tab. 1) and functional annotation of the 2,519 corresponding DE genes identified as significantly enriched by Gene Ontology (GO) (Tab. 2) and enriched Reactome canonical pathway biological processes restricted to the 345 GO *cell cycle* associated genes (Tab. 3).

**Figure S1 : EMX2 expression in U251 transfected cells.**

A- Production of a tetracycline-regulated *EMX2* expression system in U251 cells. Experimental design. Six distinct, stable, double transfected clones were constructed. First, U251 cells were transfected using the regulatory vector pcDNA6/TR. The two resulting clones (TR cl. C and TR cl. D) were further transfected with pcDNA4/TO/*myc-*HisA_*EMX2*. We selected three stable clones derived from each regulator clone, TR cl.C (EMX2 cl.C.1, EMX2 cl.C.2 and EMX2 cl.C.3) and TR cl.D (EMX2 cl.D.1, EMX2 cl.D.2 and EMX2 cl.D.3).

B-C- *EMX2* expression in the tetracycline-inducible system. *EMX2* mRNA levels in distinct clones: six independent clones were used (the three clones derived from the regulator clone TR cl.C and the three clones derived from the TR cl.D). *EMX2* mRNA level was measured at day 0 (no induction) and 24 hours after tetracycline-induction (B). Welch Two Sample t-test on EMX2 cl.C. **(**J 24 hours versus no Tet p=0.01128) and on EMX2 cl.D. **(**J 24 hours versus no Tet p=0.001439). EMX2 protein levels after 24 hours of culture with (+) or without (-) tetracycline-induction in empty clones (Control) and two independant EMX2 clones (C).

D- Cell proliferation was measured in three different culture conditions over 31 days without tetracycline (No Tet-black line), with tetracycline (Tet-dotted line) and with tetracycline for the first 8 days only (D8 Tet-gray line). Distinct triplicate experimental cultures were performed for empty vector and for three EMX2 clones (EMX2 cl.C.1, EMX2 cl.D.1 and EMX2 cl.D.2). Proliferation curves were statistically validated using a linear mix model stratified on the batch effect (see Materials and Methods).
